# Burnout in Surgical Trainees: a Narrative Review of Trends, Contributors, Consequences and Possible Interventions

**DOI:** 10.1007/s12262-021-03047-y

**Published:** 2021-07-29

**Authors:** Judith Johnson, Tmam Abdulaziz Al-Ghunaim, Chandra Shekhar Biyani, Anthony Montgomery, Roland Morley, Daryl B. O’Connor

**Affiliations:** 1 School of Psychology, University of Leeds, Leeds, LS29JT UK; 2 Bradford Institute for Health Research, Bradford Royal Infirmary, Bradford, BD96RJ UK; 3 School of Public Health and Community Medicine, University of New South Wales, Sydney, 2052 Australia; 4 Department of Urology, St James’s University Hospital, Beckett Street, Leeds, LS9 7TF UK; 5 Cadaveric Simulation Programme, Anatomy Department, School of Medicine, University of Leeds, Leeds, LS2 9JT UK; 6 Department of Educational and Social Policy, University of Macedonia, Thessaloniki, Greece; 7 Imperial College Healthcare NHS Trust, London, W2 1NY UK

**Keywords:** Burnout, Surgeons, Surgical training, Workforce, COVID-19, Patient safety

## Abstract

Surgical disciplines are popular and training places are competitive to obtain, but trainees report higher levels of burnout than either their non-surgical peers or attending or consultant surgeons. In this review, we critically summarise evidence on trends and changes in burnout over the past decade, contributors to surgical trainee burnout, the personal and professional consequences of burnout and consider the evidence for interventions. There is no evidence for a linear increase in burnout levels in surgeons over the past decade but the impact of the COVID-19 pandemic has yet to be established and is likely to be significant. Working long hours and experiencing stressful interpersonal interactions at work are associated with higher burnout in trainees but feeling more supported by training programmes and receiving workplace supervision are associated with reduced burnout. Burnout is associated with poorer overall mental and physical well-being in surgical trainees and has also been linked with the delivery of less safe patient care in this group. Useful interventions could include mentorship and improving work conditions, but there is a need for more and higher quality studies.

## Introduction

Surgical specialties are popular and the application process for obtaining a training place continues to be competitive [1–3], despite growing interest in non-surgical specialties such as radiology [2, 4]. Students and interns who are attracted to surgical careers are influenced by surgery’s prestige, skilful nature, intellectual challenge and the ability to improve or save lives [5, 6]. However, specialising in surgery is also associated with high levels of burnout, the psychological syndrome characterised by feelings of emotional exhaustion and disengagement or ‘depersonalisation’ [7]. Evidence from a recent systematic review and meta-analysis of 89 independent studies indicates that surgical trainees report higher levels of burnout than their non-surgical peers [8]. This has also been found in a study in India, where surgical residents scored significantly higher than anaesthesia residents on several burnout indicators relating to neglect of personal needs [9].

The COVID-19 pandemic has placed unprecedented levels of stress on healthcare professionals across disciplines, health sectors and countries [10, 11]. Even prior to the COVID-19 pandemic, there was evidence of high levels of work-related stress and burnout in physicians overall. In the UK, a 2018 survey of 51,956 trainee doctors found that 1 in 4 felt burnt out to a high degree or very high degree [12], and in the USA, a 2019 study of 4893 physicians indicated that approximately 44% were experiencing at least one symptom of burnout [13]. There are several questionnaires used to measure burnout, such as the Maslach Burnout Inventory [14], Oldenburg Burnout Inventory [15] and Copenhagen Burnout Inventory [16] which conceptualise and measure burnout in varying ways. Furthermore, these questionnaires are often adapted in individual studies to make them shorter [e.g. 17,18]. Due to this variation in measurement tools, direct comparison of results between studies can be challenging and it is hard to identify whether there are real differences in burnout rates between countries [19]. However, it is clear that burnout is a common occupational health problem in physicians internationally, which is a concern to all medical disciplines, including surgery.

The research findings now emerging from studies conducted during the pandemic indicate that it has had differential effects upon surgeons’ well-being depending upon various factors, including which subspecialty they are in and whether they are trainees [11, 20]. These have included both work factors such as the physical discomfort of wearing personal protective equipment (PPE) and the psychological stress of managing a frequently changing work pattern alongside personal factors such childcare problems due to school closures and an inability to see family and friends [11]. Surgical trainees have been particularly vulnerable to the impact of the pandemic, which has reduced educational and career development opportunities, leaving some anxious about passing their annual reviews [11, 20]. The long-term impact of the pandemic on the mental health of surgeons and surgical trainees is currently unclear but evidence from previous pandemics indicates that this will likely be long-lasting [21]. Enduring effects could include elevated symptoms of post-traumatic stress and increases in smoking and drinking alcohol [21]. As such, there is an urgent need to better understand the causes and consequences of burnout in surgeons to inform the development of supportive interventions. There is also a particular need to understand these issues in surgical trainees, who have been significantly affected by the pandemic and whose retention is of crucial importance in supporting the delivery of healthcare services moving forward.

## Trends

**Fig. 1:**
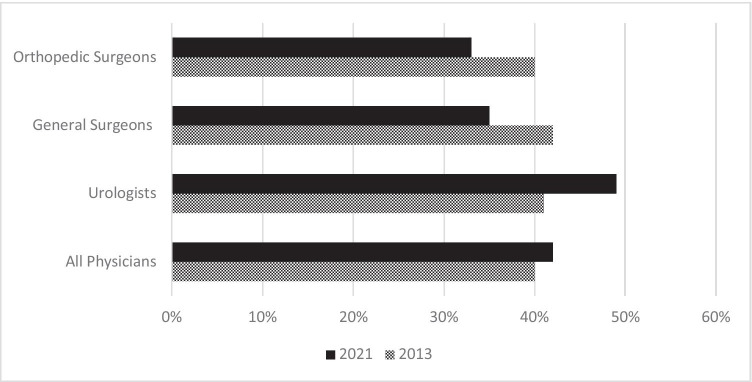
Percentage of US orthopaedic surgeons, US general surgeons, US urologists and all US physicians reporting burnout in 2013 and 2021

Studies into burnout have proliferated over the past decade and the number published has increased exponentially from 2014 onwards [22]. This growing literature has allowed for the identification of broad trends and patterns which can help provide context to understanding studies into burnout in surgeons and surgical trainees. First, it seems that levels of burnout have been largely stable in physicians over time. The 2013 Medscape survey found that overall, 40% of physicians reported experiencing burnout; in 2021, the rate had only increased to 42%, despite the added stress of the COVID-19 pandemic [23, 24]. In general surgeons and orthopaedic surgeons, the same survey indicated a small drop in burnout over the same time window (Fig. 1) but a comparable increase in urologists where the rate increased from 41 to 49% [23, 24]. The absence of a widespread upwards trend in burnout in physicians more generally and surgeons in particular has also been corroborated by other surveys [13]. It should be noted though that this data has all been drawn from US physicians and may not reflect patterns of burnout in other countries. Furthermore, while no evidence indicates a steady increase in burnout over the past decade, data on overlapping constructs (e.g. healthcare professional stress levels) suggests that there is likely to have been a real-terms reduction in staff well-being due to the COVID-19 pandemic [25].

We are not aware of longitudinal studies which focus on surgical trainees and so must extrapolate from wider evidence suggesting that it is unlikely that burnout has increased in a linear trend in this group over recent years. The apparent stability of levels of burnout seems counterintuitive given a growing awareness of burnout as a problem. These null findings could potentially be attributed to weaknesses in the measurement of burnout, but similar trends have also been found in studies of other mental health indicators such as depression [26]. For example, a 2015 meta-analysis in resident physicians estimated a year-on-year increase of 0.5% in depression prevalence, but the authors suggested that this might be explained by wider community cohort changes or growing awareness of depression, rather than real increases over time [27].

Instead, it has been suggested that rather than burnout increasing over time, the greater awareness of burnout has been driven by changing attitudes, with younger cohorts of medical students prioritising personal well-being and work-life balance more highly [28, 29]. Moreover, while awareness of burnout was growing prior to the COVID-19 pandemic, it has subsequently been further prioritised following the pressures on healthcare services and focus on healthcare professional well-being elicited by the crisis [25]. Given the increased levels of burnout present in surgical trainees relative to trainees in other medical specialities [8], it is possible that this trend could reduce the desirability of specialising in surgery and so threaten the sustainability of the surgical workforce moving forwards. Indeed, a recent survey of general surgery residents in the USA found that 40% would not choose surgery again [30] and a survey of orthopaedic and trauma surgery (OTS) residents in France reported that 61% would not recommend OTS to their children [27].

There is evidence that this trend may already be impacting application patterns, with news reports from India suggesting that dozens of surgery training places went unfilled in 2017 and 2018 [31]. A less marked but continued decline in popularity of surgical training places is also evident in other countries, including the USA and UK. For example, one questionnaire study reported that general surgery was significantly less preferred as a first choice among 2015 UK medical graduates compared with those graduating between 2005 and 2009 [4]. This difference was found in male students rather than female students, with only 22% of males stating surgery as their first choice in 2015 compared with 30% in the 2005–2009 cohorts [4].

One clear finding is that surgical trainees are higher risk for burnout than consultant or attending surgeons. This pattern has been reported both within original studies directly comparing residents and consultant or attending surgeons and in systematic reviews comparing results between studies. In a survey of US Orthopaedic Surgery Residents and Faculty, it was found that 56% of residents reported burnout compared with 28% of faculty members [32]. In a systematic review of 41 studies across surgical specialties, high levels of depersonalisation were found in 46–53% of residents compared with 7–26% of attending surgeons and high levels of emotional exhaustion were found in 17–42% of residents compared with 9–31% of attendings [33]. This pattern is not unique to surgical residents and has been observed in post-graduate medical trainees across specialties [8], including in a recent study of residents and faculty members from multiple specialities in India [34]. However, surgical residents consistently exceed medical norms for burnout [8, 33].

The observation that burnout is higher in trainees overlaps with findings indicating higher levels of burnout in younger surgeons than older surgeons more broadly [35]. It could be suggested that this may be a confounding factor and that it is not training per se that increases burnout. However, the association between age and burnout appears to be less robust than the association between training status and burnout [35]. Furthermore, the observation that features of training programmes, such as the presence of formal mentoring, are associated with lower levels of burnout refutes this suggestion [36].

## Contributors to Burnout

### Training Programme Factors

Studies have linked higher burnout in surgeons with their overall attitude towards their training programme, reflected in poorer ratings on programme satisfaction [37] and their likelihood of recommending their programme to others [38]. Links have also been made with specific aspects of training programmes. A recent study in US plastic surgery residents found that higher burnout was not linked with age, gender, race or relationship status but was linked with feeling less involved in programme-related decisions [38]. Similarly, a survey of US general surgery residents found no links between burnout and gender or relationship status, but did report that burnout was higher in residents reporting fewer programmatic social events and lower in residents receiving programme mentorship [30]. Links between receiving mentorship and reporting lower burnout have also been reported in other studies of surgical trainees [32, 39, 40]. However, there is no evidence to suggest that burnout varies consistently according to training stage, with studies showing mixed and variable findings in burnout rates across training years [32, 36, 39, 41].

### Work Factors

Higher burnout has been linked with greater number of working hours overall [38, 40, 42, 43] and working more weekends per month [30]. One study in 665 US general surgery residents found that 69% of respondents were burnt out, and those reporting high burnout worked on average 3 h more per week than those reporting low burnout [40]. Associations between workload factors and burnout have not replicated consistently, with some findings indicating no association between number of work hours or the delivery of night shifts and burnout in trainees [44, 45], and a study from Saudi Arabia reporting no significant association between number of on-calls per month and emotional exhaustion in plastic surgery residents [46].

Burnout has been found to be lower when surgery trainees report the presence of more work support factors. For example, in plastic surgery residents in France, lower risk of burnout was associated with the occurrence of regular staff meetings and weekly ward rounds by senior surgeons [44]. In a study of US general surgery residents, lower risk of burnout was linked with autonomy at work, feedback, perceived social support and opportunities for development [41]. Conversely, factors which could generate a sense of being unsupported have been associated with higher risk of burnout. In a study of digestive surgery trainees in France, it was found that perceiving a lack of gratitude from senior staff, an insufficient amount of practical training and an overly high level of responsibility were all associated with a greater risk of reporting burnout [47].

Colleague factors are also important to burnout. Studies have found that burnout is lower in surgery trainees when they report being friends with their co-workers [48] and when they feel a sense of social belonging in their specialty [49]. Conversely, burnout is higher when trainees report negative colleague interactions, such as poor interactions with nurses [50], being stressed by relationships at work [32] or being shamed and humiliated at work, for example by being banished from the operating room or being called ‘stupid’ [51].

### Personal Characteristics

Gender is frequently tested in relation to burnout in consultant and attending surgeons, with an overall conclusion that burnout risk is higher in women [52]. However, the picture is less clear in surgical trainees. While some studies have reported that burnout overall [41, 43] or the emotional exhaustion facet in particular is higher in women [40, 49], this finding has not always replicated. Several studies have reported no association between gender and either emotional exhaustion [53] or overall burnout [27, 30, 38] and other studies have highlighted that burnout overall [45] or the depersonalisation facet is in fact higher in men [40, 53]. As undergraduate medical degrees are increasingly taking higher proportions of female students [54] but surgical training remains a less popular choice among this group [4], understanding links between gender and burnout and making improvements based on this will be important for the sustainability of surgical disciplines in the future.

Of all the studies which have investigated associations between burnout and personality characteristics in surgeons, the majority of these have been in trainee groups [35]. These have suggested that higher emotional intelligence [41, 55, 56] agreeableness, conscientiousness and emotional stability [41], grit, resilience [57, 58] and dispositional mindfulness [53] are associated with lower burnout. However, the investigation of personality in surgeons and surgical trainees is contentious; the usefulness of such knowledge is unclear and some commentators have highlighted its potential for misleading and deterring prospective surgical trainee candidates [59].

A range of studies have investigated family factors but findings have been mixed. While being single has been identified as a risk factor for burnout in attending and consultant surgeons [35] and this has occasionally replicated in studies of surgical trainees [27, 50], several studies have reported no association between relationship status and burnout in trainee groups [38, 40, 44, 60]. Furthermore, having children was found to be associated with lower levels of burnout in a study of surgical residents in Pakistan [45] but this has not always replicated [40, 44]. Similar with studies into personality variables, it could be suggested that understanding links between family factors and burnout has limited use and could potentially stigmatise individuals based on characteristics which are not related to their work and which they may have limited latitude to change.

### COVID-19 Pandemic

Research clearly indicates the influence of the COVID-19 pandemic on surgeon burnout: in a UK survey, 86% of consultant and trainee surgeons reported negative effects related to the pandemic, including emotional exhaustion [11]. In the Netherlands, surgeons assigned to a COVID ward reported high burnout symptoms [61]. In India, 1 in every 2 orthopaedic surgeons reported experiencing either mild, moderate or severe anxiety in May 2020 [62]. However, further research is needed which focuses on surgical trainees; this group may be particularly vulnerable to burnout given the pandemic’s impact on their training and education [11].

## Consequences of Burnout

### Personal Consequences

Broader reviews of surgeons have identified burnout as an associate of numerous detrimental personal consequences, including a higher risk of reporting a mental health disorder and poorer physical quality of life [35, 63]. In surgical trainees in particular, burnout is associated with higher levels of depression [27, 53, 64, 65], anxiety [53], PTSD [66] and suicidality [53]. One study reported no association between burnout and suicidality in US general surgery trainees, but the sample size was relatively small (*n* = 92) and, given the rarity of suicidal thinking, may be attributable to lack of statistical power [64].

Burnout has also been associated with unhealthy behaviours in surgical trainees such as alcohol misuse [32] and engaging less frequently in exercise [32, 46, 64], although these findings have not always replicated [44, 48]. While it could be suggested that burnout is a contributory factor to these symptoms and behaviours, most studies have been cross-sectional and so it is not possible to establish causality. Instead, it may be more useful and accurate to view burnout as a warning sign which could indicate that a wider pattern of poor mental health and unhelpful behaviours could be present. This is supported by studies linking higher burnout to poorer self-reported general health [3], physical health quality of life [32, 48] and happiness levels [48].

### Patient Care Consequences

The first systematic review investigating associations between burnout and patient safety in healthcare professionals was published in 2016 [67]. Across the 30 studies which were included, 21 (64%) reported a significant association between burnout and patient safety outcomes [67]. Since this time, this research field has flourished with numerous further original studies being reported. This growth has enabled the subsequent publishing of reviews focused on specific professional groups such as physicians and primary healthcare providers [68, 69]. It has also allowed for a wider range of patient care outcomes to be synthesised including provider communication and quality of care indicators [68–70]. In India, higher burnout in doctors has been associated with poorer communication with patients, including being more likely to shout at patients [34].

No systematic review has investigated the association between burnout and patient care outcomes in surgeons or surgical trainees specifically, but results from a limited number of original studies in surgical trainees reflect findings in the wider literature. For example, in US plastic surgery residents, significant associations were found between higher burnout and having made an error that could have resulted in patient harm or a lab error [38]. There was a trend towards burnout being associated with medication errors but no association was found between burnout and having made an error that did result in patient harm [15]. In OTS residents in France [27] and in US abdominal transplant surgery fellows, higher burnout was associated with a greater risk of having made a medical error in the past 3 months [48]. In a study of US general surgery residents, it was found that overall burnout and each of the emotional exhaustion and depersonalisation facets were significantly associated with a higher self-reported risk of having made a medical error that resulted in harm, and making a near-miss that did not result in harm [71].

In terms of surgeons and medical error, one area of research that may be particularly important is the relationship between burnout and cognitive impairments [72]. For example, two studies observed significant associations between burnout and cognitive decline only in complex cognitive tasks whereas performance on less complex tasks was within the normal range [73, 74]. Interestingly, burnout is associated with poorer performance on cognitive tasks tapping attention and visuo-spatial constructional ability [75]. Additionally, a study revealed that healthcare professionals (doctors, nurses, psychologists) with stress-related exhaustion take longer to switch their attention between tasks [76]. Moreover, research with internal medicine residents and faculty members show that high depersonalisation scores were associated with a decreased blood oxygenation level dependent (BOLD) effect in the right dorsolateral prefrontal cortex (dlPFC) and middle frontal gyrus, while high exhaustion scores were associated with more right posterior cingulate cortex and middle frontal gyrus BOLD, three brain regions that are associated with executive functions, memory and attention, respectively [77]. Error monitoring impairments, as indicated by event-related potential (ERP) patterns, have also been observed among burnout populations [78]. Disrupted ERP patterns indicative of a high cognitive workload have also been observed among surgical residents [79]. Overall, the impact of burnout on cognitive functioning most relevant to surgical practice (i.e. attention, executive functions and visuo-spatial abilities) is likely to have negative impacts on patient safety.

Together, these findings indicate that surgical resident burnout is likely to be associated with patient care outcomes. However, these have been cross-sectional and mainly conducted in the USA; further research is needed to establish the direction of causality between burnout and patient care outcomes, to examine whether this is present in other countries and to investigate a wider range of outcomes. This literature has also been criticised for an over-reliance on self-reported rather than objectively measured errors [35].

## Burnout Interventions

While being higher in burnout than physicians in other specialties [8], surgeons are also less likely to seek help than other medical professionals [80]. This is particularly concerning when it is considered that physicians have higher rates of suicide than individuals in other occupations, and that surgeons have one of the highest rates of suicide of all medical professionals [81]. As such, it is of crucial importance to consider both (1) which interventions may be beneficial for reducing burnout and improve mental wellbeing in surgeons and (2) how these interventions can be designed and delivered in such a way to enhance uptake among surgeons. Given that surgical trainees have higher rates of burnout than consultant and attending surgeons, these considerations are particularly relevant in this group.

Interventions for burnout are usually divided into those which are ‘person-directed’ and those which are ‘organisation-directed’ [82]. Person-directed interventions focus on supporting the individual with the problems they are reporting and include mindfulness and employee-assistance programmes [83, 84]. Organisation-directed interventions take a more contextual approach, viewing the individual as a worker in the context of their workplace and aim to ameliorate burnout by addressing workplace stressors, for example by improving working hours or conditions. They can also include interventions which aim to provide workers with more job-related support, such as workplace training or mentoring [85].

Organisation-directed interventions have increasingly been favoured by academic commentators as the more logical and acceptable approach [86]. However, several issues make this challenging to consider. First, it is unclear whether organisation-directed interventions are indeed more effective than person-directed interventions [82, 87, 88]. Second, many interventions do not neatly fit into either the ‘person-directed’ or ‘organisation-directed’ categories and may involve multiple aspects spanning these categories [82, 88]. Third, due to challenges in implementing and researching organisation-directed interventions to the standards required by academic journals, there is an overall lack of published studies on these intervention types.

### Interventions in Surgical Trainees

It can be concluded from the existing literature that interventions, overall, are effective for reducing burnout in physicians [82, 88], but effect sizes are small and there is a need to improve these. We are not aware of any systematic review that investigates burnout reduction interventions in surgeons or surgical trainees in particular, but in a 2017 review of burnout reduction interventions in residents across specialties, a total of 19 original studies were identified including 6 randomised controlled trials and 13 cohort studies [89]. Of the included studies, 3 were in surgical specialties and 1 was in obstetrics and gynaecology residents, and 9 focused upon the 2003 and 2011 Accreditation Council for Graduate Medical Education duty hour restrictions [89]. Overall, the review found that the duty hour restrictions were associated with reductions in emotional exhaustion and depersonalisation. They also reported limited evidence supporting meditation and self-care workshop interventions [89].

A small number of original interventional studies have been published since the publication of this review which aimed to reduce burnout in surgical trainees and measured this as an outcome. In a controlled study of 21 US surgery residents, mindfulness meditation training was associated with post-intervention improvements in perceived stress and mindfulness in the active group (*n* = 12) compared with the control group (*n* = 9) but no differences were observed for burnout [90]. However, it must be noted that the sample size for this study was small and null findings could be attributed to a lack of statistical power. In a cohort study of 8 Canadian otolaryngology-head and neck surgery residents, a formal mentorship training programme was associated with reductions in emotional exhaustion and depersonalisation at 12-month follow-up, but as there was no control group, it is possible that this could be attributed to the effects of time [91].

### Future Directions

There is a clear need for more and higher quality studies testing burnout interventions in surgeons and surgical trainees. Most studies to date have been cross-sectional, had a small sample size or have not included a control sample, which prevents firm conclusions being drawn regarding intervention effectiveness. There has also been a preponderance of research from the USA, and there is a need for studies which are conducted in other nations, particularly in low- and middle-income countries (LMIC). Despite this, several promising avenues for future research can be identified. Findings from studies of risk factors related to surgical trainee burnout suggest that changes made to training programmes could be useful targets for reducing burnout and could be manageable to implement, such as the introduction of formal mentorship [30]. Changes to work conditions, such as improving working hours, have also been reported to have tangible benefits for burnout [89]. Such interventions can be distributed widely to all trainees prophylactically, which could reduce the risk of trainees choosing not to engage due to the stigma of reporting burnout.

The intense and demanding nature of surgical work promotes high levels of identification within departments and units—creating the phenomenon of small organisations (i.e. surgical units) within the bigger one (i.e. hospital). One approach that is particularly suited to comparing across units is the areas of worklife model, which specifies six areas in which the job-person match is critical: workload, control, reward, community, fairness and values [92]. It a useful framework for identifying possible ways to improve the job environment by enabling focus on problems that people care about and are willing to work on, until there is an improvement—which then can cross-pollinate to other areas. For example, organisations where staff have set their own working patterns (affecting the areas of fairness and control) have shown improved recruitment and staff satisfaction [93]. An intervention approach that focuses on ‘what can be done’ is possibly more likely to fit with the ethos and mindset of pragmatic surgery staff.

Furthermore, it is possible to draw on the wider literature when identifying potentially useful burnout interventions. For example, there has been a growing awareness of the contribution of technology advancements and associated technology-related frustrations to increasing healthcare professional burnout [94]. However, a recent systematic review of 81 studies in physicians found that interventions which optimise technologies, such as by providing training, reducing the time spent on electronic documentation or improving workflow processes were associated with burnout reductions [94]. These interventions can be simple to identify, design and deliver, and could become increasingly beneficial in a time where medical and surgical interventions are becoming more and more digitised.

Another interesting intervention to consider is yoga [95]. An increasing evidence base supports a link between physical health and burnout, but in a systematic review of 11 articles, yoga was associated with lower levels of stress and burnout in healthcare workers [95]. However, it should be noted that only seven of the studies included in the review were controlled trials, and there is a need for further, higher-quality evidence to gather more evidence and identify which forms of yoga may be most acceptable and effective [95].

## Conclusion

Surgical trainees experience higher levels of burnout than both their non-surgical peers and attending or consultant surgeons. Higher rates of burnout have been associated with poorer personal well-being and a greater risk of involvement in medical errors in this group. A range of factors are known to increase the risk of burnout, but interventions focused on improving work conditions by limiting work hours or increasing support through mentoring and supervision may be useful targets for interventions to focus upon. However, most of the studies into interventions for surgical trainee burnout suffer from significant methodological limitations and there is an urgent need for further, higher-quality research in this area.

## Declarations

### Disclaimer

The views expressed in this publication are those of the authors and not necessarily those of the National Institute for Health Research or the Department of Health and Social Care.
